# DEDICATE: proposal for a conceptual framework to develop dementia-friendly integrated eCare support

**DOI:** 10.1186/s12938-018-0552-y

**Published:** 2018-09-12

**Authors:** Sara Marceglia, Michael Rigby, Albert Alonso, Debbie Keeling, Lutz Kubitschke, Giuseppe Pozzi

**Affiliations:** 1 Dipartimento di Ingegneria e Architettura, Università degli Studi di Trieste, Via A. Valerio 10, 34127 Trieste, Italy; 2 Fondazione IRCCS Ca’ Granda Ospedale Maggiore Policlinico, Milan, Italy; 3 Health Information Strategy, Keele University, Keele, UK; 4 Hospital Clinic Barcelona, Barcelona, Spain; 5 University of Sussex, Brighton, UK; 6 Empirica Communications and Technology Research, Bonn, Germany; 7 Dipartimento di Elettronica, Informazione e Bioingegneria, Politecnico di Milano, Milan, Italy

**Keywords:** Dementia, Integrated care, Integrated eCare, Patient-centred, Family-centred, Care infrastructure, eHealth, ICT architecture, Socio-technical ecosystem

## Abstract

**Background:**

Evidence shows that the implementation of information and communication technologies (ICT) enabled services supporting integrated dementia care represents an opportunity that faces multi-pronged challenges. First, the provision of dementia support is fragmented and often inappropriate. Second, available ICT solutions in this field do not address the full spectrum of support needs arising across an individual’s whole dementia journey. Current solutions fail to harness the potential of available validated e-health services, such as telehealth and telecare, for the purposes of dementia care. Third, there is a lack of understanding of how viable business models in this field can operate. The field comprises both professional and non-professional players that interact and have roles to play in ensuring that useful technologies are developed, implemented and used.

**Methods:**

Starting from a literature review, including relevant pilot projects for ICT-based dementia care, we define the major requirements of a system able to overcome the limitations evidenced in the literature, and how this system should be integrated in the socio-technical ecosystem characterizing this disease. From here, we define the DEDICATE architecture of such a system, and the conceptual framework mapping the architecture over the requirements.

**Results:**

We identified three macro-requirements, namely the need to overcome: deficient technology innovation, deficient service process innovation, and deficient business models innovation. The proposed architecture is a three level architecture in which the center (data layer) includes patients’ and informal caregivers’ preferences, memories, and other personal data relevant to sustain the dementia journey, is connected through a middleware (service layer), which guarantees core IT services and integration, to dedicated applications (application layer) to sustain dementia care (formal support services, FSS), and to existing formal care infrastructures, in order to guarantee care coordination (care coordination services, CCS).

**Conclusions:**

The proposed DEDICATE architecture and framework envisages a feasible means to overcome the present barriers by: (1) developing and integrating technologies that can follow the patient and the caregivers throughout the development of the condition, since the early stages in which the patient is able to build up preferences and memories will be used in the later stages to maximise personalization and thereby improve efficacy and usability (technology innovation); (2) guaranteeing the care coordination between formal and informal caregivers, and giving an active yet supported role to the latter (service innovation); and (3) integrating existing infrastructures and care models to decrease the cost of the overall care pathway, by improving system interoperability (business model innovation).

## Background

Dementia is recognised as a growing societal health and care problem due to the increasing survival of older people in society [1, 2]. Not only does dementia cause serious problems for the individual concerned, but it also severely disrupts and stresses the lives of family carers, neighbours, and others inevitably involved in supporting a person with dementia [3]. At the same time, services in most countries and localities across Europe are fragmented and far from adequately developed [4]. However, economic and personnel constraints are such that re-engineering of service delivery into smarter services is at least as important as increased investment. As such, the state of the art of support for dementia in the majority of locations in Europe is seriously unsatisfactory [5]. Alongside fragmentation and silos within and between care agencies, there are weak interfaces with societal support, and the generally limited support available is dispensed to carers according to availability. Yet, there is a strong recognition of the potential and value of use of information and communication technologies (ICTs) to improve the quality of life of patients with dementia and Alzheimer’s, and, in turn, that of their carers [3, 6]. Evidence from earlier pilot projects points to ICT-based integration of care services for older people as resulting in improved outcomes, client satisfaction and efficiency gains [7]. In particular, it is envisaged that ICTs will enable future systems to recognize and support the needs of caregivers, who take care of mostly (highly) dependent dementia patients, thus reducing stress and depression with concomitant long term benefits [3].

To enable such potential, there is the need to define a clear reference model and conceptual framework including all the main requirements targeted to dementia patients, and including formal and informal carers. For this reason, in this paper we propose a conceptual framework and a possible architecture that could be used to develop effective ICT-based solutions for dementia patients, that we called DEDICATE. Starting from a state-of-the-art research aimed to identify the main factors hampering a full exploitation of ICT-based solutions for dementia; we ideated a socio-technical model designed for addressing the need for user-supporting and user-driven ICT-enabled services to coordinate integrated patient-centric health and care to those with dementia, from the early stages of diagnosis to full severity. Starting from the socio-technical model, we propose an architectural approach aimed to guide the implementation of future systems supporting the full formal and informal care team, as well as the patients.

It may be unusual to public a complete conceptual model ahead of prototyping and piloting. However, we feel there are deficiencies in the current normal developmental models—namely, either proprietary product development protected against widespread early review; or research grant proposals which are secret, and which normally cannot be amended between submission for consideration and final completion of a grant-funded project. Both these approaches limit constructively critical discourse, lock development into one route, and lead to defensive attitudes to evaluation at the end point [8]. They also make difficult and expensive end-stage modification. This paper aims to enable open discussion ahead of construction, to enable a better outcome and putting societal gain above solution protection.

## Methods

Since the purpose of this paper is to propose a conceptual framework and a possible reference architecture for ICT-based solutions for dementia care, we first conducted a literature research, aimed to drive the requirement definition.

The literature search was based on two sources: first, the Pubmed/Medline medical bibliographic database, second, the European Commission Portal and the Google search engine. This choice allowed opening the research not only to scientific literature but also to ongoing and past projects addressing dementia care. In the case of the Google-based general search, the results were included if they:

- Fully described the implementation of a project (or a set of projects) related to dementia care or providing a report on the state-of-the-art of dementia care (minimum date: 2013).
- Referred to the European area, in order to focus on a more homogeneous environment.

In the case of literature search, we included only reviews published after 2013 and up to 2016, in order to guarantee up-to-date the conclusions.

We identified from the literature a list of the main factors that acted as barriers to the development of effective infrastructures. From these factors, we extracted the list of requirements and the reference socio-technical ecosystem needed for dementia care [3–5, 9–12]. From them, we derived the reference architecture and the conceptual framework for its dynamic evolution. Finally, we provided a preliminary evaluation of available tools that could be used to implement a system based on the proposed architecture.

## Results

### Barriers to development of effective infrastructures

From our literature research we found several projects developed in Europe for the purposes of dementia care: ISISEMD (http://vbn.aau.dk/en/projects/isisemd(e5e57897-c1e6-4993-a668-b758d4a503cc).html), Dem@Care (http://www.demcare.eu), In-MINDD (http://www.inmindd.eu/), InnovateDementia, and STAR. These initiatives focus on assisted living, prevention regimes and neurodegenerative disease, which, though promising, only address individual aspects of the many problems, and do not provide an integrated delivery of all forms of care to cognitively impaired patients and their carers.

We then identified seven reviews/reports specifically addressing the problem of integrated dementia care across Europe, and reviewing the current status of development [3–5, 9–12]. These analyses underlined that the dangers of closed silo service provision have been recognised in some countries at the policy level, and that steps were taken to spread responsibility more widely and to introduce cooperative structures, including third sector and citizens’ groups [4].

From the searched documents we identified the following factors that hamper the mainstreaming of ICT-based solutions:

- Deficient technology innovation: recent development of ICT platforms for more flexible care delivery to older people (e.g., UniversAAL) and those having dementia (e.g., ISISMED) lack technology features and functionalities that enable integrating the full spectrum of formal and informal caregivers into a single information loop with a view to enabling truly joined-up support, including people with dementia themselves as co-producers of well-being and independent living. Long-term care for people with dementia also comprises health and social care services–diagnostic and continuing care services. However, these services are today split into organisational clusters, separately managed, delivered and recorded. At best, people with dementia and their families tend to be surrounded by uncoordinated ‘Islands of Excellence’, when what is needed is person-centered coordinated care [9].
- Deficient service process innovation: in the care domain, ICT-based services tend to be delivered within socio-technical systems, and value is frequently achieved by people (e.g., care professionals, family carers) utilizing technology for their purposes (i.e., delivering people services) not by technology alone [10]. As such, ICT-based solutions for integrated dementia care require technology innovation and service process innovation to be pursued in parallel. This includes the need to recognize co-contributions by informal carers alongside formal carers as part of one system, with due identification of the role of each and for sharing of selected appropriate information and recording. The latter aspect has, however, not as yet enjoyed sufficient attention in most pilot projects in the field of ICT-based elderly care [9]. The family always has a central role, supported to a greater or lesser extent by formal professional or para-professional care services [11]. Informal family care, representing the cornerstone in almost every country, is sometimes supported or supplemented by paid home caregivers, respite opportunities and palliative end-of-life care. Strengthening the capabilities of family carers to enable better coping with the challenges of their experience is essential [12]. Caregiver interventions, such as education and counselling, have been proven to reduce or delay transition from home into a care home [3].
- Deficient business model innovation: when it comes to the desired up-scaling of successfully piloted ICT-based solutions in the field of dementia care, evidence shows that—apart from benefits shift occurring between different parties involved in joined-up care delivery—current care systems are not always favourable to the financing of integrated eCare solutions [5, 10].

### Requirements and socio-technical ecosystem definition

Following the identification of the main barriers, we identified the following requirements, mapped to specifically address them:

#### Requirements for overcoming deficient technology innovation

- Adopting a clearly user-driven, choice-giving approach and avoiding all technology ‘push’, aiming to increase respect, quality and also efficiency and effectiveness of ICT solutions. Lifestyle choices are paramount at any age, yet hitherto these have been difficult to accommodate. A person’s preferences for mealtime patterns, foods, time of rising and going to bed, morning or evening shower or bath, music preferences and television viewing patterns are fundamentally important to sustaining confidence and independence. There is currently a strong likelihood that the pattern of care becomes provider-driven. Thus, at the very time that the person is vulnerable and needing help, their lifestyle is changed around them. This both reduces respect and increases confusion, even in the person’s own home.
- Utilising components to support persons with dementia, particularly linked to earlier diagnosis and active support to enable continued living at home and increase quality of life and comfort. ICT solutions for coordination and communication should play an effective part right from the beginning. Timely diagnosis, at a point where the person with dementia can make informed lifestyle choices, is essential but a consequence is that early support services are necessary to support the person and their family in these decisions [13]. If no early services are readily deployed, there will be no encouragement towards early diagnosis, perpetuating the current problem that diagnosis only occurs once the effects are beginning to take deeper hold. Also, denial of the diagnosis, or turning away from facing it, is not uncommon amongst people who are experiencing significant cognitive changes [14, 15].
- Including elements of telecare and telehealth where needed, respecting the wishes and preferences of the person with dementia, especially in the early stages when they should be able to express informed choices on the way they view their future care. Then, record and information presentation mechanisms need to be in place that keep knowledge available and actively refreshed, in a way that is in line with the individual’s persona and expectations. Institutional and personal memory is important for efficiency but also for sustaining independence and well-being. Over time, patients experience a loss of understanding and consistency on the small issues that facilitate acceptance of help and quality of life. This is also due to changes in their carers: informal carers may change as a result of the burden of caring, and formal carers may change through staff attrition, replacement and rosters.

#### Requirements for overcoming deficient service process innovation

- Utilising a shared planning and communication record and function across the formal and informal carer team for the individual, interfacing with legacy systems based on open protocols. Support services need to address the wider care challenge by including the social environment from the beginning, and enabling informal carers to play a fruitful role as part of an informal–formal virtual team, based on their needs, abilities and preferences [16].
- Utilising care coordination applications to run holistically as a virtual system on formal and informal care platforms (including mobile devices), such as scheduling, automatic messaging, and voice and video telephony support. Considerable improvements can be achieved by interfacing different ICT technologies to empower the remote carer [17]. The combination of video call contact with the frail relative, coupled with access to an ongoing log of carer comments, and the ability to input to that communication system, could still effectively bring a remote carer into the virtual team delivering real care.
- Planning of support in a shared timeline, linked to patient and carer needs and service capacity.

#### Requirements for overcoming deficient business models innovation

- Supporting stakeholder centric, evidence-based business case modelling. A sustainable care model for ICT-based dementia support has to be comprehensive and coordinated between the different stakeholders involved, and operated on a user-friendly platform. Integration of delivery of care services is essential for better quality, better efficiency and effectiveness, and to accommodate growing numbers within constrained resources [6, 9].
- Supporting evidence-based decision-making on the up-scaling of implemented solutions implying the inclusion, from the design phase, of appropriate information necessary for establishing the benefits and drawbacks of the solution, in line with the concept of “Evidence-Based Medical Informatics” [18].

A final unavoidable requirement is interoperability, which is considered as a prerequisite for successful service development and sustainable service operation. To this end, interoperability cannot be only associated with the ability of telecommunications and digital systems—and the processes they support—to exchange data and to enable the sharing of information and knowledge [19], but also with non-technical perspectives (i.e., organizational, policy, governance, and legal perspectives), such as that defined by the European Union eHealth Interoperability Framework [20].

**Fig. 1:**
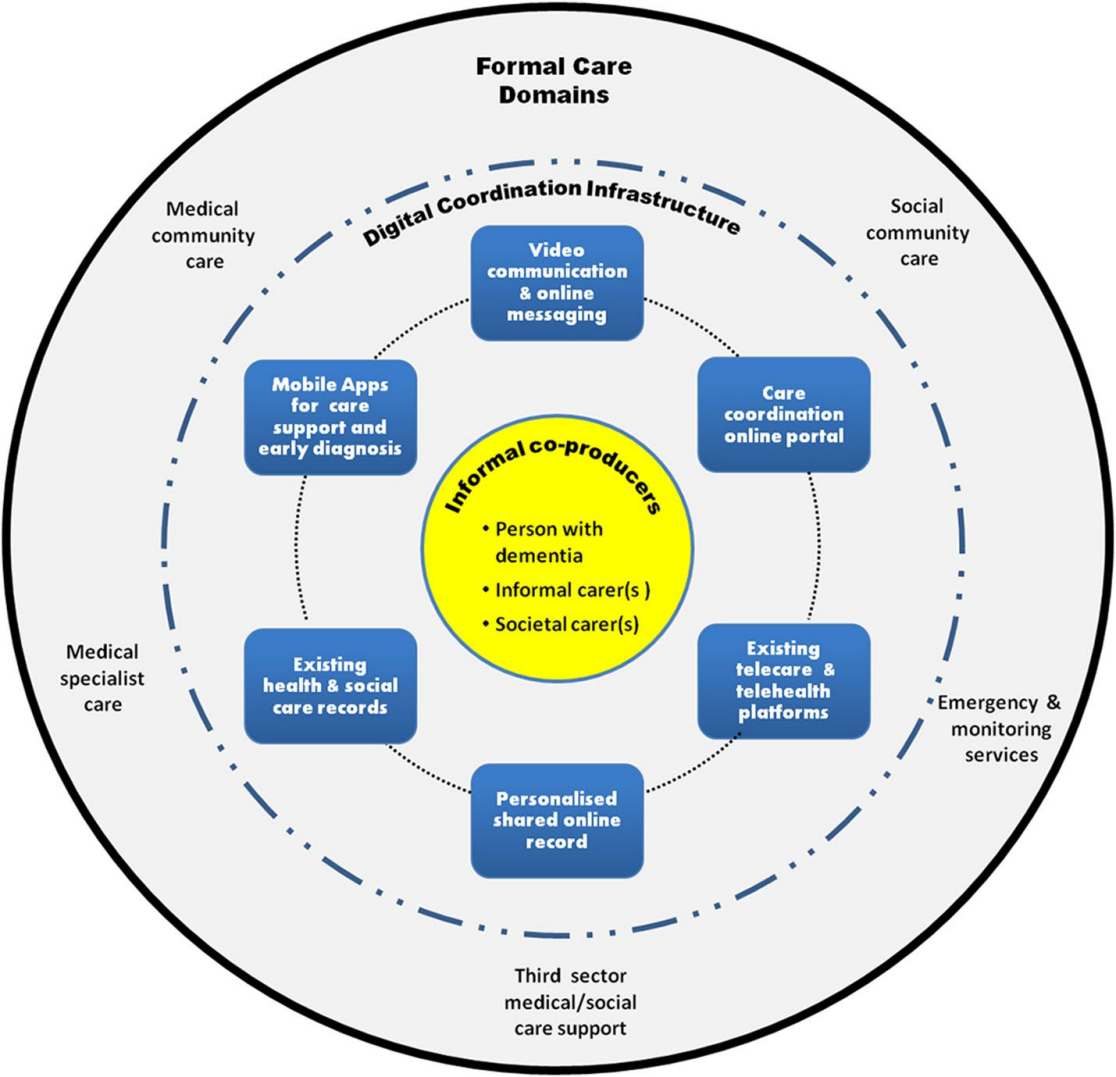
The DEDICATE socio-technical ecosystem for integrated dementia care. The patient and the family carer are the center of the ecosystem. The inner circle represents the different ICT services and functions to support both patients and their families. The different services are integrated through a coordination infrastructure (dashed circle) that allows the interaction between patients/families and healthcare professionals (external circular layer)

Taken together, these requirements suggest that any ICT-based solution aimed at delivering care to dementia patients is part of a socio-technical ecosystem (Fig. 1) that includes all relevant stakeholders as actors, uses existing systems and services, and manages needs and tools that have to be coordinated. The center of the ecosystem is composed by the patients and their informal/social caregivers (informal co-producers), whereas the borders of the ecosystem are the other care stakeholders and the existing services, representing the domain of formal care. The integration technology (digital coordination infrastructure) acts as a “sharing bus” connecting the dedicated systems addressing all the needs of the informal co-producers to the other actors (formal care domain). Technology innovation requirements, service process innovation requirements, and business model innovation requirements concur to implement such holistic ecosystem.

### Proposed architecture and conceptual framework

**Fig. 2:**
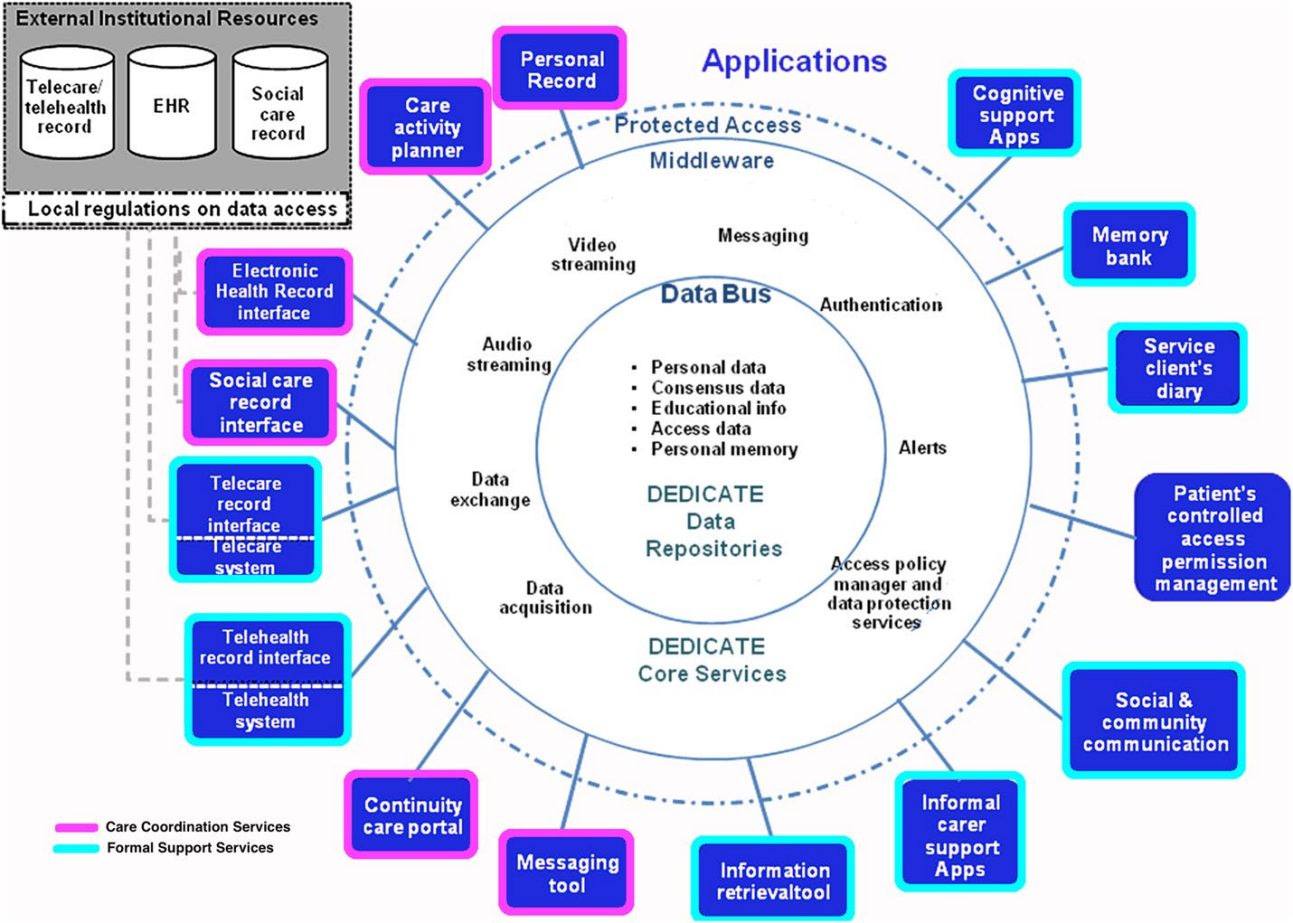
The DEDICATE integration architecture. The three-layer architecture is represented in concentric circles. The core is the data layer that is connected to the core DEDICATE service layer through a data bus. A middleware, protected by a security boundary, integrates all the applications exposed in the most external layer. The applications are targeted to the different stakeholders and dialogue with existing health record systems

Figure 2 shows the proposed architecture, which relies upon a concept of integration of different applications mediated by a middleware that introduces an abstraction layer in the system architecture and thus reduces the complexity considerably.

In general, the proposed architecture is classically based on three layers, the data layer, the service layer, and the application layer. The data layer consists in a secure storage system devoted to the management of personal preferences, educational information, and all those data needed to both longitudinally follow the patient’s journey with dementia and to guarantee informal caregiver inclusion. The service layer provides a set of core IT services that enables the main functionalities (e.g., access, audio/video communication, messaging, data acquisition/exchange). Acting as a middleware for integrating the various systems and applications in the final layer, a Service Oriented Architecture (SOA) design approach should be implemented for this layer. SOAs ensure that available services can be used by multiple different systems from several business domains and that the interaction among services is not affected by the service implementation. Hence, to guarantee interoperability, services should be designed with implementation-independent interfaces, and by implementing communication protocols that stress location transparency. Also, services should use explicit interfaces to define the encapsulated functions, so that either the service can be used by one or more systems that participate in the architecture or the service can be easily substituted by another providing the same functionalities as declared in the interface. Finally, the application layer is composed both by existing systems, including web applications and telemonitoring and telecare systems, and newly-conceived applications (e.g., exercises for cognitive support, apps for the creation of personalized memory banks, patient’s diaries, social and community applications, applications dedicated to informal carers, etc.).

The applications connected to the DEDICATE architecture can be divided into two main categories: the formal support services (FSS) and the care coordination services (CCS).

FSS aim to provide a number of support tasks to be achieved and supported throughout the various stages of the dementia journey. When dementia has been identified, there is a lot of new information to assimilate and a lot of decisions to be made. This is a period of change and upheaval both practically and emotionally for the person diagnosed and for their family and friends. At an early stage, the person themselves may become less able to recall important current information so having a reliable interface where this can be shared and not lost is crucially important (e.g., personally constructed memory banks). Also, telecare and telehealth has an increasing role to play as dementia progresses. The need for on-going support and feeling connected to favourite places and past-times remains crucial for the person living with dementia and the family carer. More specifically, FSS should:

- Provide people with information personalised to their needs at the various stages of dementia (early stage: creation of the memories; later stages: use of the memories to recall information).
- Help people with dementia and their families in adjusting to diagnosis (later stages).
- Support lifestyle changes that would help people in the longer term (later stages).
- Support people with dementia and their family in making plans and records that would help in the future (early stage).
- Support people with dementia and their families in enjoying life (all stages).
- Provide support when it comes to symptoms control and keeping fit and healthy (later stages).
- Help in dealing with crises and managing the unexpected (later stages).
- Risk management (telecare) and health monitoring (telehealth) (all stages).

CCS are, on the other hand, dedicated to the number of actors needing to coordinate their caring task(s) around the individual’s needs throughout the different stages of the dementia journey. CCS support inter-organisational cooperation at the “back office” level when it comes to organisations involved in professional care (e.g., health care and social care) and those providing non-professional support (e.g., third sector organisations). Moreover, CCS also enable efficient collaboration with the family carer. Not least, they would empower persons with dementia themselves—according to their mental faculties—to take part in effective management of their condition and maintain their independence as far as possible. Such abilities have often been overlooked [13]. More specifically, CCS should provide:

- Integrated data access for care providers in different agencies and informal carers.
- Planning, scheduling and reporting of care activities enabling temporal coordination between provision steps taken by care providers in different agencies and informal carers.
- Access to analytics for home-based monitoring data (telemonitoring and/or telecare) by care providers in different agencies and informal carers.
- Real-time communication between care providers in different agencies and informal carers, for example, support to case conferences.
- Joint response to ad-hoc requests by care providers in different agencies and informal carers.
- Self-management, including links into all above cooperation mechanisms.

The architecture hypothesizes two types of access policies. One controlled by the patient that regards the access to his/her personal information for which he/she has the full responsibility (i.e., memory bank contents, cognitive supporting apps results, etc.). One depending on the local constraints and regulations regarding health-related information controlled by the care providers (i.e., therapies and treatments, care pathways, etc.).

**Fig. 3:**
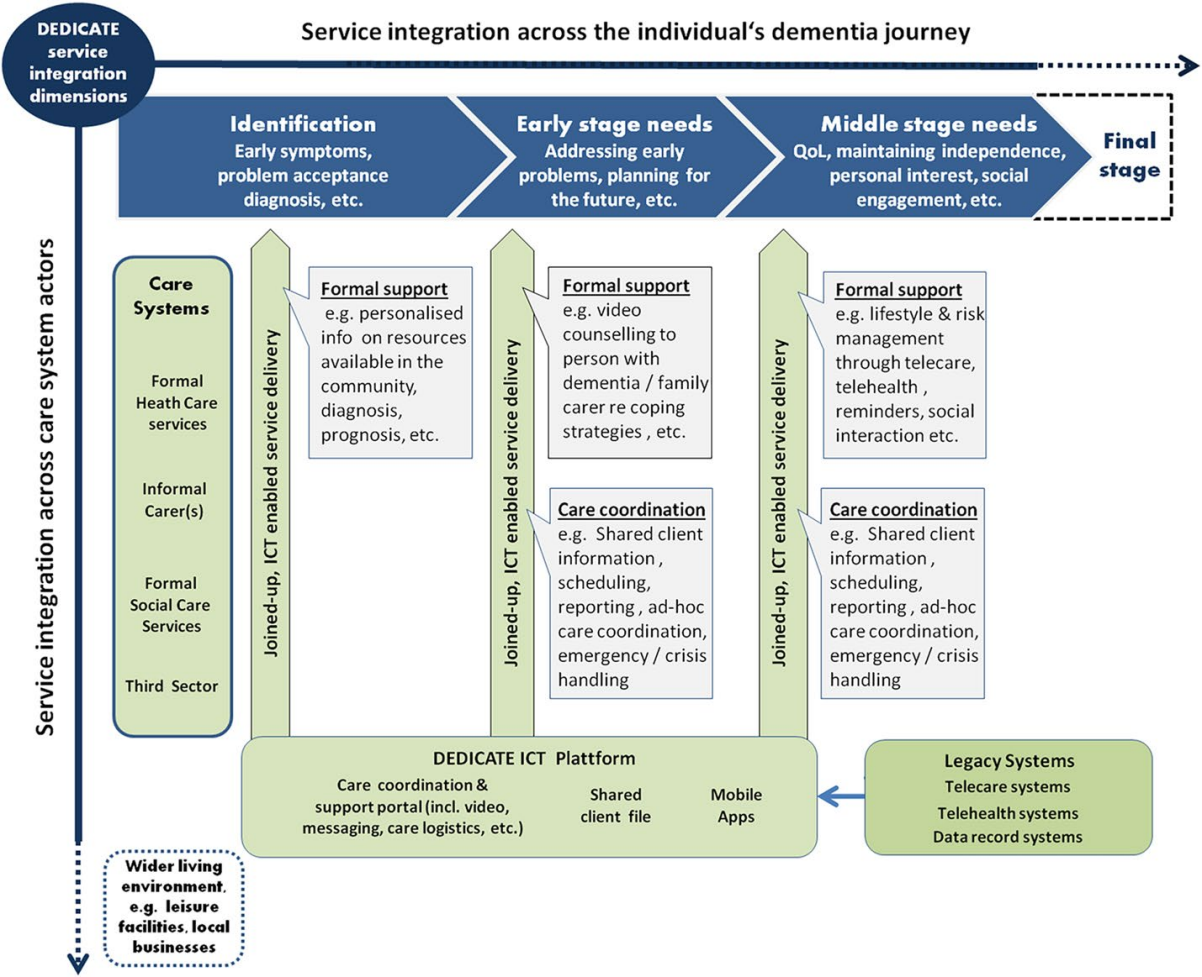
The DEDICATE architecture conceptual framework for dynamic evolution. The horizontal axis represents the dementia journey with four main stages, namely identification, early stage, middle stage, and final stage. The blue arrow represents the needs at each stage, with specific focus on the first three stages (the final stage needs are dashed). The vertical axis represents the main actors of the dementia care process, namely healthcare, family care, social care, and third sector. The vertical arrows represent the different ICT services (for direct support and/or for care coordination) that respond to the needs at each disease stage, which are integrated through the ICT platform in which the existing telecare/telehealth system are also integrated

Figure 3 illustrates the conceptual framework that shows how the proposed architecture fulfills the expected requirements. More specifically, the three macro-requirements are achieved through a threefold service integration strategy (Fig. 3).

#### Technology innovation

The DEDICATE architecture service delivery is integrated across changing needs throughout the individual’s ‘dementia journey’ (horizontal axis, in Fig. 3) that characterizes progressive dementia at different stages. Depending on the stage, different types of information and resources tend to be helpful to people with dementia and the surrounding living environment [13]. For example, when an individual has been diagnosed, this person and her/his immediate family and friends need to come to terms with the diagnosis and integrate it into their life. They may not be focused on the practicalities of managing the condition on a daily basis. In the DEDICATE architecture, this is achieved by guaranteeing, on the one hand, the management of personal preferences, memories, etc., together with the delivery of FSS that are mainly needed in early stages, and, on the other hand, adding CCS and new FSS based on the personal preferences previously created in the later stages. In fact, although it is difficult to predict how each person will fare with dementia, there is usually a point where the person begins to need significantly more help and assistance to function on a day-to-day basis at home, and, at this point, CCS should be added for improved care coordination.

#### Service innovation

The architecture guarantees integration across different key actors typically involved in dementia care (vertical axis, Fig. 3). Such integration is acknowledged as a complex process [21]; yet solutions often ignore the opportunities for incorporating the informal carer(s) into formal processes [22]. In the DEDICATE model, today’s organisational care silos are overcome by enabling coordinated cross-sector delivery of support to people with dementia. In doing so, a dual role is assigned to informal family carers. On the one hand, they receive supportive services from formal health and social care providers (e.g., counselling) enabling better coping with their caring experience. On the other hand, they are enabled to coordinate their own caring activities with formal health and social care services available in the community. Recognising the value that proactive informal carers can bring to the care journey in acting as a knowledgeable and local coordinator of care (within their stated abilities and preferences).

#### Business model innovation

The DEDICATE architecture allows the integration of new care-coordination technology and social technologies with existing home monitoring and safety technology (DEDICATE ICT Platform, Fig. 3). If newly developed ICT-based dementia services are to be enabled for up-scaling and replication, attention should be given to the considerable diversity across countries and regions in the framework conditions within which pilot services mainstreaming occurs. This situation largely reflects today’s realities of the developing market place for integrated eCare solutions [23]. Against this background, a simplistic approach to the adoption of a new model for integrated dementia care can easily be (mis)interpreted as the wholesale migration to new service processes and ICT platforms. In reality, however, such an approach poses major budgetary problems for service providers who have already invested in particular care technologies addressing at least parts of the wider spectrum of user needs arising throughout the ‘dementia journey’ (e.g., mainstream telecare systems), as well as feared disruption of services which are trusted even in their imperfection. Also, such an approach introduces risks in terms of system delivery and potential loss of service and data continuity. Therefore, the integration approach proposed in the DEDICATE architecture can introduce the benefits of a holistic approach while maintaining a controlled use of resources.

In Table 1 a detailed description of how the DEDICATE architecture and framework address the specific requirements is provided.

**Table 1:** Detailed matching between requirements, DEDICATE architecture features, expected benefits, and proposed metrics

Macro-requirement	Specific requirement	DEDICATE architecture feature	Expected benefit	Possible example metrics
Requirements for overcoming deficient technology innovation	Adopting a clearly user-driven, choice-giving approach and avoid all technology ‘push’	Build patient’s preferences and memories in early stage of disease that will remain during the entire journey. DEDICATE also envisages the use of existing technology already available to patients/families	Improved compliance to the system and long-term use	# patients using the system along the whole dementia journey Time spent (%) using the system without caregiver help Time spent (%) by the caregiver using the system
	Utilising components to support persons with dementia	Specifically developed applications are integrated to the system through the middleware to guarantee support		
	Including elements of telecare and telehealth where needed	Existing telehealth and telecare systems are integrated in the architecture		
Requirements for overcoming deficient service process innovation	Utilising a shared planning and communication record and function across the formal and informal carer team	Electronic Health Record, Social Care Record, telehealth/telecare record are all interfaced to the system, and their data are integrated in care coordination plans	Improved inclusion of healthcare professionals in the dementia journey and facilitated disease management from a medical perspective	# of unscheduled access to the healthcare system % of healthcare documents integrated in the system # access to the system by healthcare professionals to review patients’ situation
	Utilising care coordination applications to run holistically as a virtual system	Care coordination applications are part of the whole system and are supported by the core ICT services run on the service layer		
	Planning of support in a shared timeline	The architecture envisages the co-participation of all actors, from formal to informal carer as well as other stakeholder, which share the same information and plans		
Requirements for overcoming deficient business models innovation	Supporting stakeholder centric, evidence-based business case modelling	The architecture is designed so that all information is shared among stakeholders, and existing systems are integrated and not replicated	Improved sustainability and auditability of the services	# trials regarding the effectiveness of ICT interventions for dementia care based on the system % data collected by the system used for effectiveness assessment/quality assessment
	Supporting evidence-based decision-making	The integration of formal care records and personal applications will allow collecting relevant data needed to track the effectiveness of the system/interventions		

## Possible implementation scenario

The main components of the DEDICATE architecture are: a platform ensuring data management and core IT services acting as a middleware for application integration and series of dedicated applications. On top of that, existing services and infrastructures can be connected through the middleware to obtain a holistic system. The core platform can be a care coordination portal that supports the different care (‘people’) services working together with informal carers and assisted persons with dementia; rendering the ability to access a ‘CRM-type’ (Customer Relationship Management) database via a browser so that users can track, monitor and request support, or simply communicate as well as highlight where interventions and support may be required.

A possible prototype to implement the care coordination portal is the Connect-I portal, developed within the EU’s CIP programme INDEPENDENT project (http://independent-project.eu/home.html), and mainstreamed in the region of Milton Keynes, UK. This project has developed an integrated set of ICT-enabled services dealing with a range of threats to independent living common to older people, which could be specialized for persons with dementia according to the specific requirements of integrated dementia care.

Within the portal, the access to personalised information is only provided to selected individuals following consent by the care recipient. As a main strength, the Connect-I system is developed within the context of existing proven hardware and software technologies (standard PCs, operating systems, web cams, database software based on MS SharePoint Portal technologies and commonly available broadband services including open standard VOIP applications) that can be easily implemented in standard settings, without specific equipment required for patients and families. In addition, the Connect-I portal implements a wide range of functionalities, including: case record management functionalities (e.g., capability to take Notes on contact record, case management and workflow, broader ethnicity categories, etc.); reporting capabilities (e.g., attendance at a specified workshop/training/support group/event, history of a specific client, statistical breakdown of client groups, last contact date, etc.); self-book appointments for web-cam based support/help sessions, time booking system (for the caring processes); and, simple document sharing and key information library facility, events calendar and reminders system with internal messaging, telephone lists, service provider details, with ability to rank and rate by carers.

The second component is a set of mobile apps and telehealth systems for both patients and carers, supporting multiple devices, including mobile devices, such as industry standard tablet PCs, smartphones, and smart watches. For instance, a holistic care mobile health app should provide care support and health information both inside and outside the home, thus allowing the user to easily reach a carer or family member should they need to and giving autonomy for the person living with dementia and peace of mind for their carer (e.g., mHealthASSIST app, see https://www.hma.co.uk/work/featured-home-case-study/). Another exemplary app to be integrated is a cross-platform tablet app prototype to support people living with dementia in their leisure activities (digIT, see https://www.hma.co.uk/work/featured-home-case-study/), designed to engage the person as well as their carer.

Finally, the care coordination portal should be also open towards interlinkage—as far as possible based on available standards—with established record systems in the health care and social care arenas, to enable transferring person-specific health and social care-related data into the care coordination process as deemed appropriate for the purposes of integrated dementia care, thereby adhering to relevant ethics and data privacy rules/regulation.

Even though this is only an envisaged implementation of the proposed architecture, the technological elements described here are already available and can be integrated to create a real DEDICATE system. The use of familiar technologies enables the existing skills of users to be maintained and utilized, or at least, that use skills are within the realm of everyday experiences and thereby accelerating adaptation to such systems.

## Proposed evaluation and metrics

Implementing a system based on the proposed architecture and evaluating its performance and usability with respect to existing systems would be the next steps. System evaluation needs the definition of appropriate metrics, which have to be defined according to the specific case study and the priorities and values of stakeholders. However, the proposed conceptual framework can ground the identification of the outcomes and benefits that, in turn, can be used to define the metrics [24], using, for instance, the Goal Question Metrics (GQM) approach [25].

From a high-level perspective, the requirements for overcoming deficient technology innovation are expected to improve the conformity of the patients and caregivers to the system, thanks to an improved usability, acceptability, and benefits (Table 1). These benefits can be measured, for instance, by tracking the number of patients using the system along the whole dementia journey, or the percentage of time spent by the patients using the system with and without caregiver help.

Similarly, the requirements for overcoming deficient service process innovation are expected to improve the integration of healthcare professionals in the dementia journey, as well as the disease management from a medical perspective. These benefits can be measured by monitoring the number of unscheduled accesses to the healthcare system, or the percentage of healthcare documents that are successfully integrated into the system, and accessed by healthcare professionals to review patients’ situation (Table 1).

Next, the requirements for overcoming deficient business models innovation would improve the long-term sustainability of the ICT interventions, as well as their monitoring. Hence, the metrics can be used to measure the usage of the data collected through the system for evaluating the effectiveness and/or the quality of the interventions for the dementia patients (Table 1).

Finally and holistically, the core benefits can be measured in terms of patient gain, such as by length of successful living at home, reduction of adverse incidents, or reduced carer fatigue symptoms; and in terms of service provider benefit from optimized use of time, and reduced emergency calls or emergency room attendances. This matches the true aim of stakeholders for optimum quality of life, and efficient use of resources.

## Discussion

This work presents a conceptual framework and architecture for supporting the development of ICT-based solutions that overcome the presently existing barriers. The proposed approach is grounded on the principles of avoiding the fragmentation of support by focusing on holism and integration, of giving continuity along the path of disease progression, and of seeking to accommodate all supporting options, thereby also seeking to delay or even avoid institutional admission.

The proposed DEDICATE architecture is based on the pursuit of: coordinated working of formal carers and informal carers with their clients, patients and families; affordable technology infrastructure, featuring volume (‘high street’) products where possible; interoperability of services, information and products; and, the adoption of open interface standards. It also envisages the interoperability between a range of applications that can be implemented by different systems according to the different infrastructures available. Hence, the proposed architecture can be applied to different environments with different constraints, provided that the whole set of interoperability principles are fulfilled.

The value of this work resides in the definition of a complete holistic architects’ and people vision—encompassing both patients and carers—which is fundamental before building an implementation. The integration of the sociological, technical, caring, and governance issues into one vision is itself an accomplishment, and a far healthier springboard than starting with a project-led technology-based build.

The first innovative aspect introduced by the whole DEDICATE architecture is to focus on different stages of dementia in a holistic manner, rather than selected aspects emerging during the typical dementia journey at a particular stage. The real benefits of this approach will be slow to demonstrate, as they are aimed in particular at delaying—or even avoiding—permanent admission to residential facilities as well as long-term mental health issues developing amongst care groups. Meanwhile, the interim benefits should, not least, be reduced anxiety and confusion in newly diagnosed persons and their family carers, and reduced adverse incidents and emergency interventions or admissions.

Secondly, the DEDICATE architecture is innovative by introducing the idea to enable the person with dementia, with their carers and over a period of time, to build up a profile of themselves. This includes familiar events and key memories, so that these will be available to future carers to act as a means of personalisation, but it also includes a recording of preferences and wishes ranging from lifestyle preferences to wishes on patterns of care at a future more dependent stage of their journey. It also enables formal carers to document professional and institutional knowledge about the individual as a care recipient, including responses to interventions and best means of obtaining cooperation, facilitating care in the future as staff or settings change.

Thirdly, the DEDICATE architecture has been designed to move well beyond normal practice by providing a vehicle for coordination of care. There are two aspects of such coordination. One is temporal coordination, particularly in the next and later stages of the disease, enabling support to be coordinated and distributed according to need of the person and the family carers, and to times they prefer. The other one is communication and messaging facility, whereby carers can pass on messages about needs, or other issues, and have the means of communication with the formal care team, if necessary, because of an anxiety or a deterioration in the patient’s condition.

As described above, the implementation of the DEDICATE architecture does not require the development of new technologies but it introduces an innovative way for its construction and adoption. Whatever the starting solution, a range of functions, from care delivery schedules to personal profiles, and from care plans to remote monitoring results, should be bundled into the deployed platform to give a holistic application. Since users have access, including informal carers by individual agreement, this platform should provide a means whereby a next-of-kin living at a distance (even in another jurisdiction) will be able to keep in touch with the care team.

### Limitations

However, the applicability of the approach may face several limiting non-technological challenges. First, the DEDICATE architecture, while addressing the needs and requirements of patients and carers, does not take into account the regulatory and governance constraints that may be encountered during implementations in real settings and that can vary in different deployment contexts. Second, although social care provider organisations increasingly maintain electronic records bringing together all the relevant information for a service user into an electronic data repository, the present stage of deployment of electronic social care records (ESR) is less widespread when compared with EHRs, and the level of integration between ESRs and EHRs is still low. This would introduce the need to integrate the social and the health record without relying on recognized standards, thus limiting the generalizability of the solution deployed in a specific site. Nevertheless there is work internationally in different regions in identifying what would be needed to establish these standards as well an integrated care record (see, for instance, http://wiki.hl7.org/index.php?title=Coordination_of_Care_Services_Specification_Project). Harmonised overarching operational and governance supervision are essential prerequisites, but understanding and development of these is still rare. Even though new technologies are available, and the vision of a new paradigm of service benefits can be shared, the organizational and human challenges of agreeing accountability and governance are often the greatest challenges—hopefully the articulation of an achievable step change will stimulate the drive to overcome these hurdles. Lastly, we are still facing fragmented reimbursement systems to achieve integrated service financing. This applies in two dimensions. First, the funding of the services themselves, and secondly the funding and maintenance of the inter-sectoral electronic ecosystem.

Finally, there are always user issues to be considered. For example, user understanding of and trust in a secure system of access to personal information and care records. We should not underestimate the value of the information that is being committed by the individual, their family and carers into the system. Also, the emphasis on familiar, everyday technologies that build on existing or, at least, familiar skills, recognizes user skills as a pivotal issue in the successful implementation of any system and aims to accelerate skills development. For these reasons, the DEDICATE architecture is built around the concepts of family/carers/patients, and it takes into account the ‘needs understanding’ instead of the technological implementation of the system.

## Conclusions

In conclusion, the benefit of creating and describing the DEDICATE conceptual framework and architecture is that, taking a longitudinal perspective, it puts the needs of patients and carers foremost and central with technology as the enabler of a new human approach. The DEDICATE architecture should be adopted as a framework grounding the development of new ICT-based solutions for dementia care. Putting it forward for critical debate at this stage is aimed at ensuring strengthening, gaining greater understanding from the very different stakeholders necessarily involved, as well as stimulating interest in progressing development and implementation.

## References

[1] Abbott A (2011). Dementia: a problem for our age. Nature.

[2] Prince M, Wimo A, Guerchet M, Ali G-C, Wu Y-T, Prina M. World alzheimer report 2015: the global impact of dementia, an analysis of prevalence, incidence, cost and trends. London: Alzheimer’s Disease International; 2015. https://www.alz.co.uk/research/world-report-2015.

[3] Martínez-Alcalá CI, Pliego-Pastrana P, Rosales-Lagarde A, Lopez-Noguerola JS, Molina-Trinidad EM (2016). Information and communication technologies in the care of the elderly: systematic review of applications aimed at patients with dementia and caregivers. JMIR Rehabil Assist Technol..

[4] Leichsenring K, Billings J, Nies H (2013). Long-term care in Europe: improving policy and practice.

[5] Meyer I, Müller S, Kubitschke L, editors. Achieving effective integrated E-Care beyond the silos. IGI Global; 2014. 10.4018/978-1-4666-6138-7.

[6] ICTs and the Health Sector. Paris: OECD Publishing; 2013. 10.1787/9789264202863-en.

[7] Stroetmann KA, Kubitsche L, Robinson S, Stroetmann V, Cullen K, McDaid D (2010). How can telehealth help in the provision of integrated care?.

[8] Michael R (2001). Evaluation: 16 powerful reasons why not to do it- and 6 over-riding imperatives. Stud Health Technol Inform..

[9] Rigby M, Koch S, Keeling DI, Hill P (2013). Developing a New Understanding of enabling health and wellbeing in Europe: Harmonising health and social care delivery and informatics support to ensure holistic care.

[10] Stroetmann KA (2013). Achieving the integrated and smart health and wellbeing paradigm: a call for policy research and action on governance and business models. Int J Med Inf..

[11] De Cola MC, Lo Buono V, Mento A, Foti M, Marino S, Bramanti P (2017). Unmet needs for family caregivers of elderly people with dementia living in italy: what do we know so far and what should we do next?. Inq J Med Care Organ Provis Financ..

[12] Dam AEH, Boots LMM, van Boxtel MPJ, Verhey FRJ, de Vugt ME (2017). A mismatch between supply and demand of social support in dementia care: a qualitative study on the perspectives of spousal caregivers and their social network members. Int Psychogeriatr..

[13] Herron RV, Rosenberg MW (1982). “Not there yet”: examining community support from the perspective of people with dementia and their partners in care. Soc Sci Med.

[14] Joseph-Williams N, Elwyn G, Edwards A (2014). Knowledge is not power for patients: a systematic review and thematic synthesis of patient-reported barriers and facilitators to shared decision making. Patient Educ Couns.

[15] Higgins T, Larson E, Schnall R (2017). Unraveling the meaning of patient engagement: a concept analysis. Patient Educ Couns.

[16] McColl-Kennedy JR, Snyder H, Elg M, Witell L, Helkkula A, Hogan SJ (2017). The changing role of the health care customer: review, synthesis and research agenda. J Serv Manag..

[17] Piraino E, Byrne K, Heckman GA, Stolee P (2017). Caring in the information age: personal online networks to improve caregiver support. Can Geriatr J CGJ..

[18] Rigby M, Ammenwerth E. The need for evidence in health informatics. In: Ammenwerth E, Rigby M, editors. Evidence-based health informatics – promoting safety and efficiency through scientific methods and ethical policy. Studies in health technology and informatics series, vol. 222. Amsterdam: IOS Press; 2016. pp. 634–637. http://ebooks.iospress.nl/volumearticle/42811.

[19] European Community (2004). European interoperability framework for pan-European eGovernment services.

[20] European Commission, Directorate-General for the Information Society and Media (2011). European eHealth interoperability roadmap: final European progress report.

[21] Spanjol J, Cui AS, Nakata C, Sharp LK, Crawford SY, Xiao Y (2015). Co-production of prolonged, complex, and negative services: an examination of medication adherence in chronically ill individuals. J Serv Res..

[22] Ferrante S, Bonacina S, Pozzi G, Pinciroli F, Marceglia S (2016). A design methodology for medical processes. Appl Clin Inform..

[23] Kubitsche L, Cullen K. ICT and ageing—European study on users, markets and technologies; 2010. http://ec.europa.eu/information_society/newsroom/cf/dae/document.cfm?doc_id=952. Accessed 7 Sept 2018.

[24] Marceglia S, Ferrante S, Bonacina S, Pinciroli F, Lasorsa I, Savino C (2016). Domains of health IT and tailoring of evaluation: practicing process modeling for multi-stakeholder benefits. Stud Health Technol Inform..

[25] van Solingen R, Basili V, Caldiera G, Rombach HD, Marciniak JJ (2002). Goal question metric (GQM) approach. Encyclopedia of software engineering.

